# A case of left atrial intimal sarcoma with rhabdomyosarcoma differentiation: a case report and literature review

**DOI:** 10.3389/fonc.2024.1340115

**Published:** 2024-05-21

**Authors:** Hongyun Shu, Duan Xiao, Sisi Han, Yongkang Du, Jinduan Lin, Qiaowen Li

**Affiliations:** ^1^ Department of Cardiovascular Medicine, The Affiliated Qingyuan Hospital of Guangzhou Medical University, Qingyuan, China; ^2^ Department of Rehabilitation, The First Affiliated Hospital of Jinan University, Guangzhou, China; ^3^ Department of Laboratory Medicine, The Affiliated Qingyuan Hospital of Guangzhou Medical University, Qingyuan, China; ^4^ Institute of Gerontology, Guangzhou Geriatric Hospital, Guangzhou Medical University, Guangzhou, China

**Keywords:** primary cardiac tumors, intimal sarcoma, rhabdomyosarcoma, diagnosis, treatment

## Abstract

Primary cardiac malignancies are rare, with cardiac sarcomas being the main type. Among these, intimal sarcomas are the most common. However, they tend to occur in the great vessels and are rare in the heart, with only a few isolated cases reported. We report a challenging case of a patient with left atrial intimal sarcoma with rhabdomyosarcoma differentiation. The patient was admitted after a physical examination detected left heart occupancy, and initial imaging suspected a left atrial thrombus. The patient then underwent extracorporeal circulation-assisted open cardiac surgery with resection of an atrial mass. The postoperative pathological findings were suggestive of an arterial intimal sarcoma, which included areas of rhabdomyosarcoma differentiation within the tumor tissue. Unfortunately, the patient’s tumor recurred 4 months later, and she died due to treatment failure. This case highlights the rarity and risk of misdiagnosis of cardiac intimal sarcoma. Additionally, we aim to improve the understanding of intimal sarcoma through a review of immunohistochemistry and gene amplification techniques.

## Introduction

Primary cardiac tumors are rare, with an incidence of 0.001–0.03% based on autopsy results, of which approximately 25% are malignant (1). Primary cardiac malignancies lack specific clinical manifestations. Intracardiac masses can obstruct blood flow and invade cardiac valves, causing symptoms of congestive heart failure, including syncope, dyspnoea, angina pectoris, and peripheral oedema (2). Various arrhythmias may be associated with the formation of scar-like material around the sarcoma tissue, and some tumors may invade the pericardium (3).

Intimal sarcoma (IS) is an extremely rare malignant mesenchymal tumor and is also known as a spindle cell tumor, which occurs mostly in large vessels such as the pulmonary arteries and less commonly in the heart (4). Neuville et al. retrospectively analyzed 100 primary cardiac tumors and classified them according to murine double minute 2 (MDM2) amplification and found that ISs were the most common among primary cardiac malignancies (42%); they are characterized by rapid invasion into the lumen, possessing high mitotic activity (5); and because of the heterogeneous nature of IS, which, based on morphology and immunohistochemistry, is speculated to originate from subendothelial multifunctional stem cells that can differentiate into vascular, rhabdomyosarcoma or osteosarcoma histotypes, has led to the need for genetic testing, in addition to morphological and immunohistochemical tests, if necessary, in determining the final diagnosis (6, 7). Due to the rarity of these diseases, there is a lack of standard treatment and surgical resection is still the preferred option. These tumors have a high degree of malignancy, susceptibility to recurrence and metastasis, and a very poor prognosis. Here, we will describe a case of IS of the left atrium in a patient with postoperative histologic findings suggestive of IS with areas of rhabdomyosarcoma differentiation.

## Case presentation

**Figure 1:**
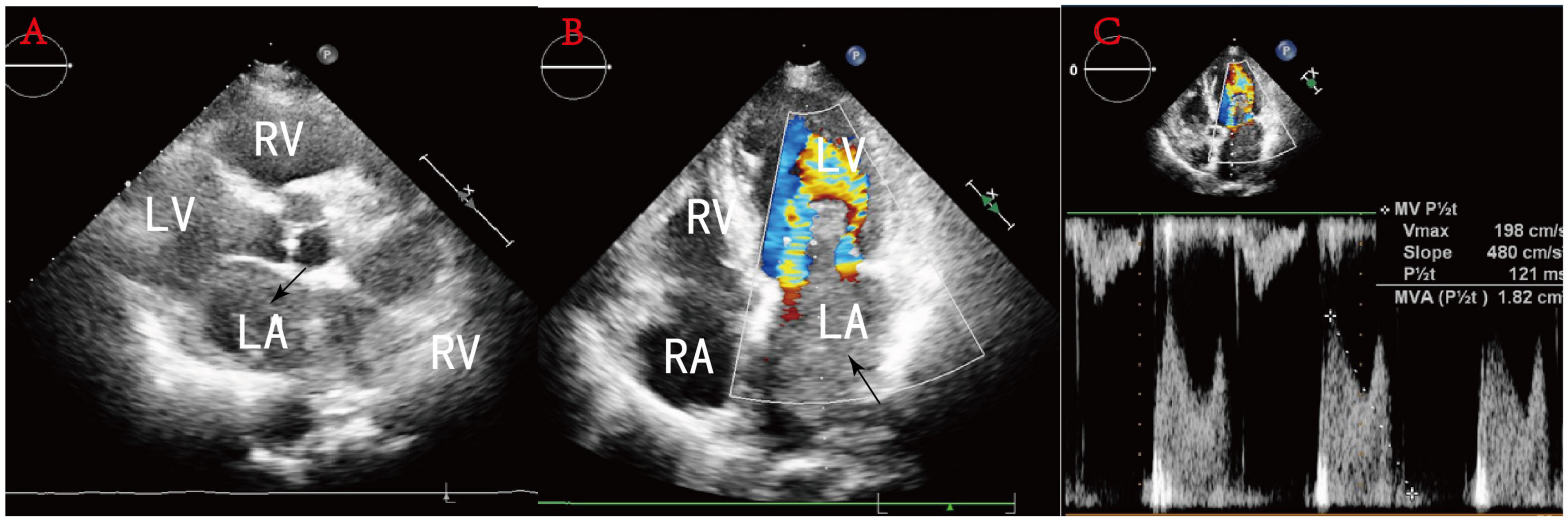
**(A)** Sternal fat left ventricular long-axis view: a hypoechoic mass is seen in the left atrium, and some of this hypoechoic mass is seen entering the left ventricle during diastole through the mitral orifice. **(B)** No obvious blood flow is seen in the mass. **(C)** Pulsed Doppler flow spectra at the mitral valve orifice: mild mitral regurgitation.

**Figure 2:**
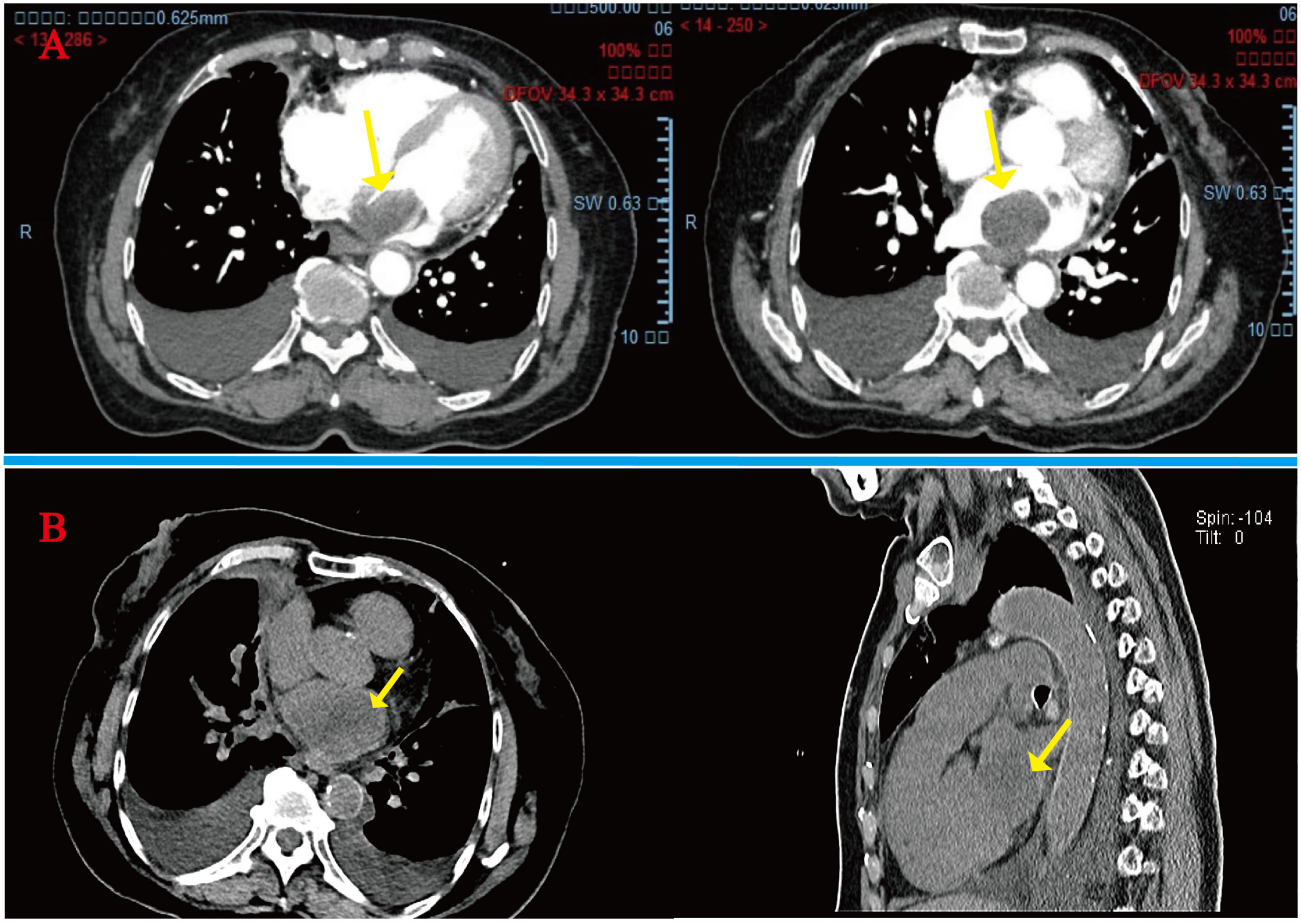
**(A)** Initial admission preoperative enhanced CT scan of the chest and abdomen: arrows indicate a homogeneous low-density mass almost entirely occupying the left atrium, extending into the left auricle and pulmonary artery opening. **(B)** Readmission CT tomography scan of the chest at 4 months postoperatively: tumor recurrence is seen at the arrows.

A 78-year-old woman presented to the outpatient clinic for “COVID-19” infection. Transthoracic echocardiography (TTE) revealed a left atrial hypoechoic mass measuring 85 mm × 55 mm × 35 mm. This mass was located in the left atrium and had a wide base connecting it to the left atrial wall. During diastole, part of the hypoechoic mass was visible, allowing blood flow to be expelled into the left ventricle through the mitral valve orifice. Additionally, mild mitral regurgitation was observed. Notably, TTE showed that the left ventricular ejection fraction (LVEF) was preserved at 68% (**Figure 1**), and the patient did not present any symptoms regarding the cardiovascular system. An enhanced computed tomography (CT) scan of the chest, abdomen, and coronary arteries on admission showed a filling defect in the left atrium, which was likewise considered to be atrial tethering (**Figure 2**). Cardiac CT and coronary angiography showed coronary atherosclerosis, including moderate stenosis of the left anterior descending branch in the middle part of the lumen and slight stenosis of the distal part of the right coronary artery. In addition, several suspicious lymph nodes were found in the mediastinum and adjacent to the great vessels. Laboratory tests revealed an N-terminal pro-B-type natriuretic peptide (NT-pro-BNP) level of 1030 pg/mL (normal range <450 pg/mL for those under 50 years old and <900 pg/mL for those over 50 years old) and a fibrinogen level of 4.88 g/L (normal range 2–4 g/L). The remainder of the admission tests did not indicate any abnormalities.

**Figure 3:**
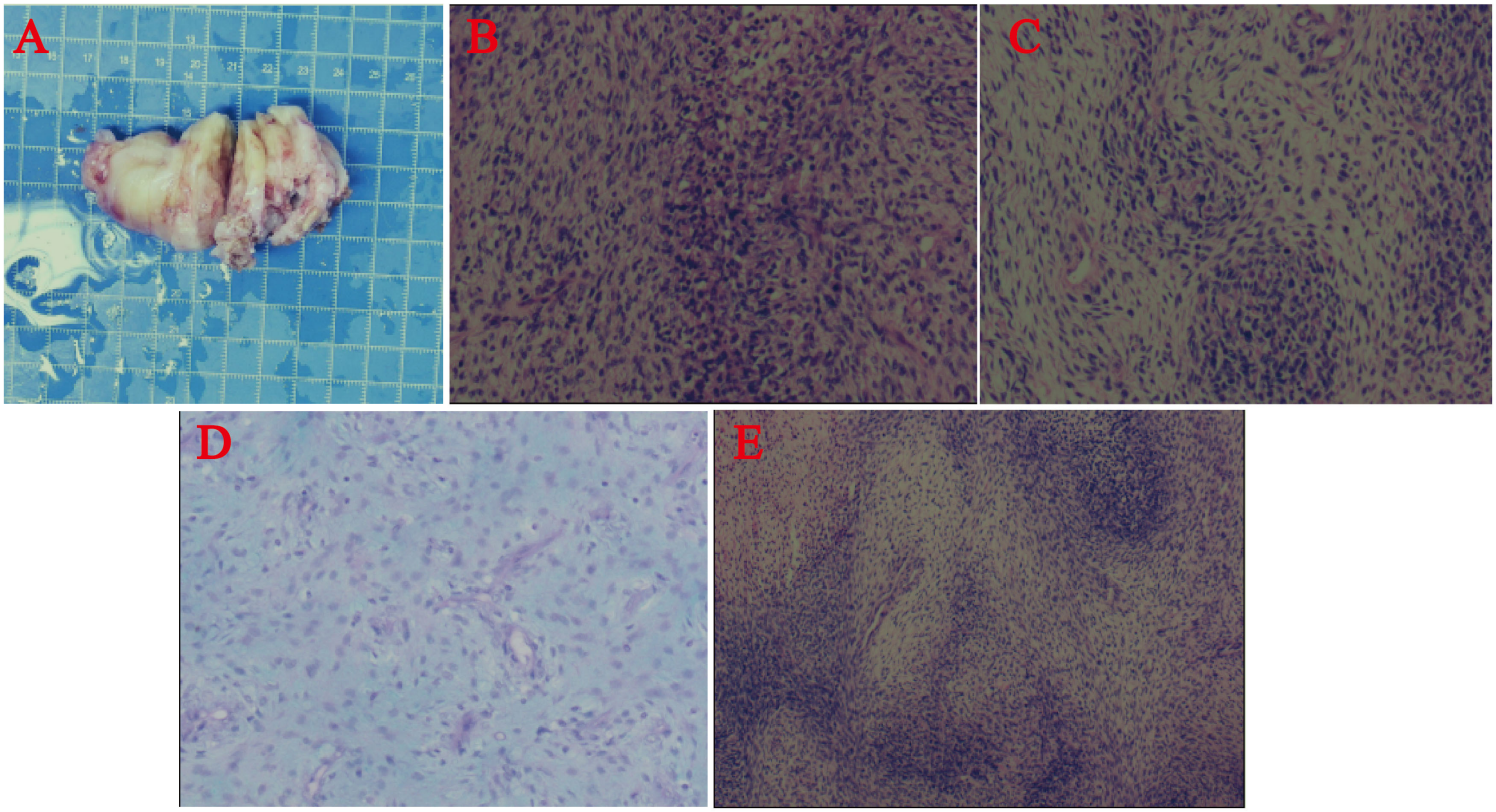
**(A)** Surgical resection of the cardiac mass. Postpathological histology and immunohistochemistry. **(B-E)** Under the microscope, the tumor was composed of dense and sparse cells, the tumor cells were spindle-shaped, with poorly defined cell boundaries, pale cytoplasm, long oval nuclei, obvious anisotropy, easy-to-see nuclear schizophrenia, and mucus-like stroma in the sparse cell area. Some of the cells are rich in cytoplasm and reddish stained, and the nuclei were biased to one side.

The patient underwent resection of the atrial tumor due to the large size of the mass and impaired valve function. During the surgery, we observed greyish-yellow and greyish-white tissue almost filling the left atrium. The mass had a solid, greyish-yellow, translucent section with focal necrosis (**Figure 3**). We performed a complete resection of the mass along its tip and used radiofrequency ablation to excise a small mass located between the pulmonary artery opening and the left atrium. No residual tumor mass was visible to the naked eye. We clipped a pericardial patch to fill the defect in the posterior wall of the left atrium. Additionally, a drainage tube was placed in the right thoracic cavity to facilitate the drainage of the pleural effusion. The endotracheal tube was removed two days after the operation, and the patient had a successful recovery. Postsurgery, bedside DR screening revealed scattered exudates in both lungs and pleural effusion without any notable features. The patient did not exhibit symptoms of heart failure, such as shortness of breath. On the 7th day after the operation, the patient was discharged from the hospital and started receiving palliative care. Further adjuvant radiotherapy and chemotherapy measures were not pursued.

In the postoperative routine pathological evaluation, the tumor was microscopically observed to consist of a dense cellular zone and a sparse cellular zone. The tumor cells had an oval shape and were clearly anisotropic, with long, oval nuclei, pale cytoplasm staining, weakly defined cell borders, and apoptosis fragmentation that was easily noticeable. Certain cells had rich, reddish-stained cytoplasm, and their nuclei were skewed to one side. In the sparse cellular zone, the visual appearance of the stroma resembled mucus. The immunohistochemistry results revealed that vimentin, CD99, CD34, CD31, and CD56 were all positive. Desmin, SMA, MyoD1, and myogenin all showed focal positive expression, although 10% of the samples had Ki67. MDM2, S-100, β-catenin, STAT6, EMA, H3K27M, H3K27Me3, P16, GFAP and CD57 all yielded negative results (**Figure 3**). The tumor was confirmed to be IS and rhabdomyosarcoma based on the immunohistochemical results and the final pathologic evaluation.

**Figure 4:**
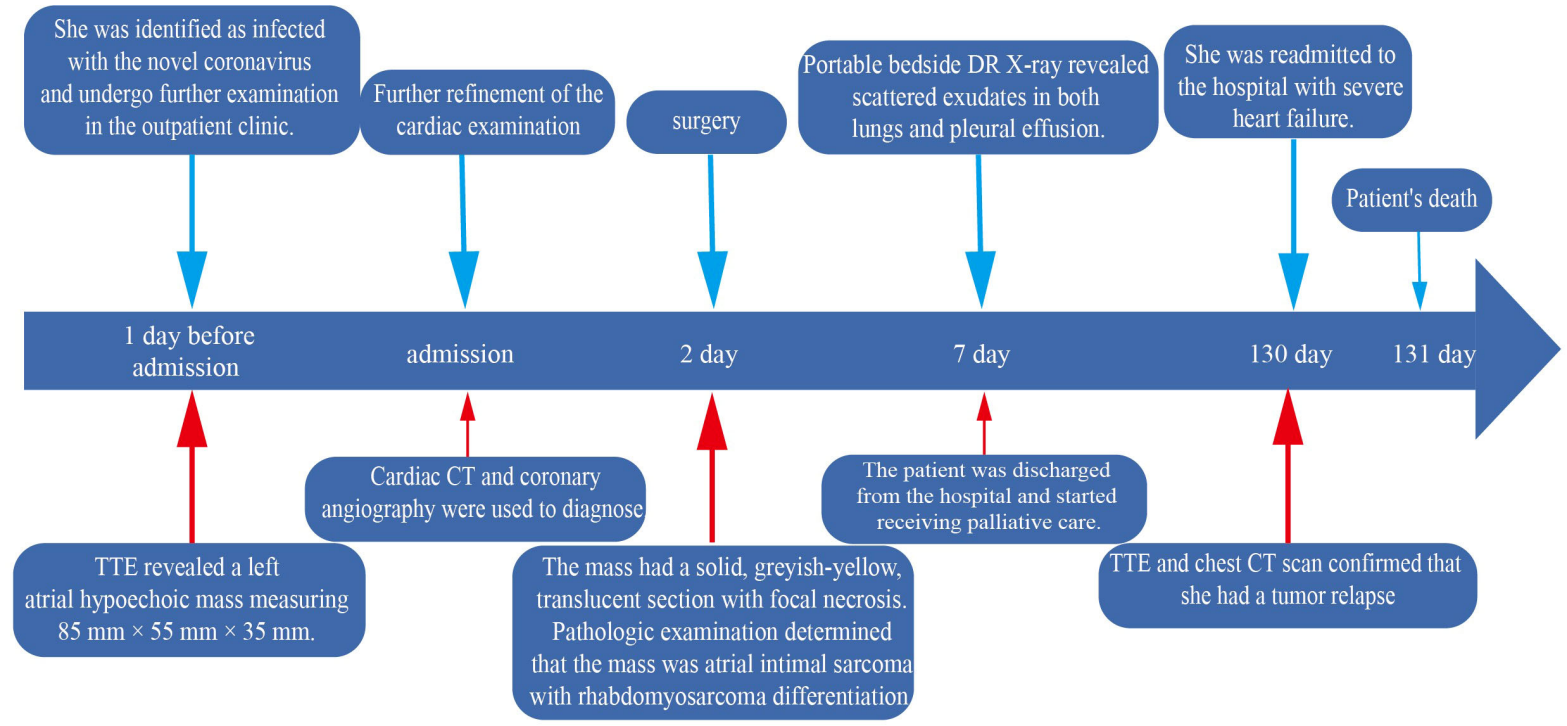
Complete timeline, including diagnosis (surgery) and treatment.

However, after the fourth month of surgery, she was readmitted to the hospital with severe heart failure symptoms, and a bedside TTE revealed a solid left atrial space causing mitral orifice obstruction, tricuspid regurgitation (moderate-to-severe), severe pulmonary hypertension, and a chest CT scan revealed a hypodense shadow in the left atrium and a large amount of pleural effusion. (**Figure 2**), We considered tumor recurrence and started urgent anti-heart failure therapy. The patient’s oxygen saturation level was extremely low, and we performed ventilator-assisted ventilation. However, on the second day after admission, the patient’s shortness of breath worsened, her consciousness appeared fuzzy, and she passed away after resuscitation (**Figure 4**).

## Discussion

Cardiac IS are rare, primarily originating in the left heart, with a lack of specificity in clinical presentation. They are often incidentally detected due to comorbidities of other diseases, typically over 40 years old. Patients with IS may be asymptomatic or have clinical manifestations of mechanical obstruction or atrioventricular block secondary to the location of the mass, and Rahmouni et al. reported a patient with IS presenting with cardiogenic shock (8). As we have reported in this article, the patient did not present with any cardiac-related symptoms at the time of her initial visit and was only accidentally found to have a cardiac occupancy on physical examination, and the TTE and CT scan revealed that an atrial thrombus was a possibility. However, the patient denied a history of atrial fibrillation, and coagulation tests did not suggest abnormally elevated levels of D-dimer, so we ruled out an atrial thrombus. The most likely diagnosis was “cardiac myxomas”, and surgery was performed for “left atrial cardiac myxomas”. After completing the histopathological examination, we established the diagnosis of IS and found areas of rhabdomyosarcoma differentiation microscopically, which was supported by the focal positivity of immunohistochemistry for MyoD1 and myogenin, which is rare in cardiac IS. The classification of similar tumors in the heart remains difficult due to the lack of diagnostic consensus among soft tissue pathologists, and the diagnosis of undifferentiated pleomorphic sarcoma versus IS is currently debatable due to the extreme similarities in their biological behaviors. However, based on the cases that have been reported and the related reports, the diagnosis of IS is preferred when the following two basic criteria are met: ① occurrence within the lumen of a large blood vessel in the pulmonary or systemic circulation or within the lumen of the heart; and ② a primary high-grade sarcoma, with or without an anisotropic component (9). Moreover, histopathological and immunohistochemical examinations are important. Immunohistochemistry of IS shows positive MDM2 expression, but MDM2 is eventually overexpressed and amplified by fluorescence *in situ* hybridization (FISH), quantitative PCR (qPCR) or array comparative genomic hybridization (CGH) to distinguish IS and other cardiac sarcoma tissue subtypes (10). Currently, the classification of cardiac sarcomas is based on histological features. Nevertheless, understanding its molecular characteristics is important for understanding the unique biology of IS and may assist in the use of molecularly targeted agents (1).Chen et al.’s preliminary investigation of 70 primary cardiac sarcomas after molecular analysis revealed potentially actionable aberrations, including MDM2 and PDGFRA amplification. Fu et al.’s analysis of 410 cancer-associated gene copy numbers from a patient with IS identified PDGFRA, MDM2, KIT genomic amplification, and CDKN2A and CDKN2B deletions in that case, confirming the emerging concept of considering PDGFR signaling as a diagnostic biomarker specific for IS (11). Tamborini sequenced the mutational hotspots of the corresponding genes and showed that these genes had no gain-of-function alterations, further supporting the notion that autocrine/paracrine loops may be responsible for receptor activation, insights that justify the use of PDGFR small-molecule inhibitors (such as imatinib, sunitinib, and nilotinib) either alone or in combination with other therapies (6).

We went through many twists and turns in determining the diagnosis. In fact, the IS is uncommon and the initial pathologic and radiologic features may be confused with those of other malignant or even benign cardiac tumors (12). Multimodal noninvasive imaging techniques play a complementary role in the preoperative diagnosis of cardiac tumors, including noninvasive and rapid TTE for the evaluation of the localization of the mass as well as the structures it invades. Typical myxomas mostly adhere to the interatrial septum with a smooth and shiny surface, and they are usually pedicled and attached to the ovale fossa (13). However, in our case, the mass observed on echocardiography was identified as being attached to the free wall of the left atrium. Furthermore, the attachment of the mass was broad, which indicates a sign of malignancy. CT can assess tissue features, vascularity, adjacent infiltrates and extracardiac metastases, and cardiac magnetic resonance imaging (MRI) is the standard diagnostic tool (14). 18F-FDG positron emission computed tomography (PET/CT) infers the presence of a malignant process based on the FDG uptake of the mass, and IS has a high FDG uptake (15, 16).

For IS, complete surgical resection is preferred (17). However, this is often impossible due to the surrounding vital structures, making tumor recurrence unavoidable, and in this case, it was rare to have such a rapid recurrence at only 4 months. In addition to this, the prognosis for IS remains very poor, with most primary cardiac malignancies having a survival of only 9 to 27 months (1). Anthracycline-based chemotherapy regimens are a potentially effective medical option for IS. Frezza’s retrospective analysis of two chemotherapy regimens in 72 patients with IS showed that the real-world overall response rate (rwORR) was 38% in the anthracycline-based treatment group (18). For patients with localized disease, the median recurrence-free survival (RFS) was 14.6 months, and for patients with advanced disease, the median progression-free survival (PFS) was 7.7 months. For gemcitabine and pazopanib, the rwORR was 8%, and the median PFS was 3.2 and 3.7 months, respectively. Because of the significant risk of radiation-induced heart disease following high-dose radiotherapy in heart radiotherapy, high-dose radiotherapy is rarely used in cardiac malignancies, forcing clinicians to adopt a reduced-dose or segmented approach to radiation therapy, Fatima et al. observed superior survival in patients who received 40–50 Gy of postoperative radiation therapy compared with surgery alone (19).

We finally clarified the diagnosis of a malignant tumor: IS, which determined the possible future treatment, although this was based on the final postpathological histology and immunohistochemistry. In our case, we have summarized several key insights: First, IS symptoms are insidious, as in our patient, who did not present with cardiac discomfort prior to surgery. Secondly, non-invasive imaging tests other than PET/CT seem to have difficulty in recognizing the features of IS. Finally, diagnosis and surgery as early as possible will provide patients with more therapeutic options, including radiotherapy and chemotherapy.

## Conclusion

Given the complexity of IS and their poor prognosis, the mass should be accurately evaluated preoperatively. Patients with atrial fibrillation are at risk of atrial thrombosis, whereas left ventricular thrombosis is associated with impaired left ventricular contraction (20). To achieve negative intraoperative margins as much as possible, when tumor resection is limited, patients with defined tumor subtypes may benefit from gene-targeted therapy with PDGFRA or MDM2, especially for those with less differentiated IS.

## Data availability statement

The original contributions presented in the study are included in the article/supplementary material. Further inquiries can be directed to the corresponding authors.

## Ethics statement

The studies involving humans were approved by The Ethics Review Board of the Sixth Affiliated Hospital of Guangzhou Medical University. The studies were conducted in accordance with the local legislation and institutional requirements. The participants provided their written informed consent to participate in this study. Ethical approval was not required for the study involving animals in accordance with the local legislation and institutional requirements because no animal experiments were performed. Written informed consent was obtained from the individual(s) for the publication of any potentially identifiable images or data included in this article.

## Author contributions

HS: Writing – original draft, Investigation, Software. QL: Writing – review & editing. JL: Writing – original draft, Writing – review & editing. DX: Writing – review & editing. SH: Writing – review & editing. YD: Writing – original draft.

## References

[1] Chen TW Loong HH Srikanthan A Zer A Barua R Butany J . Primary cardiac sarcomas: A multi-national retrospective review. Cancer Med. (2019) 8:104–10. doi: 10.1002/cam4.1897 PMC6346258 30575309

[2] Vander Salm TJ . Unusual primary tumors of the heart. Semin Thorac Cardiovasc Surg. (2000) 12:89–100. doi: 10.1053/ct.2000.5080 10807431

[3] Kumar S Barbhaiya C Nagashima K Choi EK Epstein LM John RM . Ventricular tachycardia in cardiac sarcoidosis: characterization of ventricular substrate and outcomes of catheter ablation. Circ Arrhythm Electrophysiol. (2015) 8:87–93. doi: 10.1161/circep.114.002145 25527825

[4] Durieux R Tchana-Sato V Lavigne JP Radermecker MA Moonen M Scagnol I . Recurrent cardiac intimal sarcoma misdiagnosed as a myxoma or Malignant transformation of a cardiac myxoma? J Card Surg. (2021) 36:357–62. doi: 10.1111/jocs.15200 33225534

[5] Neuville A Collin F Bruneval P Parrens M Thivolet F Gomez-Brouchet A . Intimal sarcoma is the most frequent primary cardiac sarcoma: clinicopathologic and molecular retrospective analysis of 100 primary cardiac sarcomas. Am J Surg Pathol. (2014) 38:461–9. doi: 10.1097/pas.0000000000000184 24625414

[6] Tamborini E Casieri P Miselli F Orsenigo M Negri T Piacenza C . Analysis of potential receptor tyrosine kinase targets in intimal and mural sarcomas. J Pathol. (2007) 212:227–35. doi: 10.1002/path.2177 17471466

[7] Chen D Zhu G Wang D Zhang Z Fang W Qu Z . Clinicopathological and immunohistochemical features of pulmonary artery sarcoma: A report of three cases and review of the literature. Oncol Lett. (2016) 11:2820–6. doi: 10.3892/ol.2016.4308 PMC4812211 27073558

[8] Rahmouni K Al Abri Q Janelle M Nguyen VH Sabapathy C Bernier PL . Intimal cardiac sarcoma in an adolescent presenting with sudden cardiogenic shock. Pediatr Blood Cancer. (2021) 68:e29083. doi: 10.1002/pbc.29083 33894047

[9] Cho H Song IH Jo U Jeong JS Koo HJ Yang DH . Primary cardiac sarcomas: A clinicopathologic study in a single institution with 25 years of experience with an emphasis on MDM2 expression and adjuvant therapy for prognosis. Cancer Med. (2023) 12:16815–28. doi: 10.1002/cam4.6303 PMC10501235 37395142

[10] Vinod P Jabri A Hegde V Lahorra J Cutler D . Functional mitral stenosis: imposture of primary cardiac intimal sarcoma. Cardiol Res. (2018) 9:307–13. doi: 10.14740/cr748w PMC6188046 30344829

[11] Fu X Niu W Li J Kiliti AJ Al-Ahmadie HA Iyer G . Activating mutation of PDGFRB gene in a rare cardiac undifferentiated intimal sarcoma of the left atrium: a case report. Oncotarget. (2017) 8:81709–16. doi: 10.18632/oncotarget.20700 PMC5655321 29113426

[12] Marques Mendes E Ferreira A Felgueiras P Silva A Ribeiro C Guerra D . Primary intimal sarcoma of the left atrium presenting with constitutional symptoms. Oxf Med Case Rep. (2017) 2017:omx031. doi: 10.1093/omcr/omx031 PMC5497514 28694971

[13] Griborio-Guzman AG Aseyev OI Shah H Sadreddini M . Cardiac myxomas: clinical presentation, diagnosis and management. Heart. (2022) 108:827–33. doi: 10.1136/heartjnl-2021-319479 34493547

[14] Ye N Lan L Hu H Liu J Xu H . Case report: The diagnostic challenge of primary cardiac intimal sarcoma. Front Cardiovasc Med. (2023) 10:1089636. doi: 10.3389/fcvm.2023.1089636 36844745 PMC9947778

[15] Janssen N Verheyen J Albert A . Right atrial intimal sarcoma on 18F-FDG PET/CT. Clin Nucl Med. (2020) 45:e307–8. doi: 10.1097/rlu.0000000000003051 32404706

[16] Klein MA Scalcione LR Youn T Shah RA Katz DS Sung WW . Intensely hypermetabolic lipomatous hypertrophy of the interatrial septum on 18-FDG PET with MRI and CT correlation. Clin Nucl Med. (2010) 35:972–3. doi: 10.1097/RLU.0b013e3181f9dfeb 21206236

[17] Pomp J van Asselen B Tersteeg RHA Vink A Hassink RJ van der Kaaij NP . Sarcoma of the heart treated with stereotactic MR-guided online adaptive radiation therapy. Case Rep Oncol. (2021) 14:453–8. doi: 10.1159/000513623 PMC7983626 33790766

[18] Frezza AM Assi T Lo Vullo S Ben-Ami E Dufresne A Yonemori K . Systemic treatments in MDM2 positive intimal sarcoma: A multicentre experience with anthracycline, gemcitabine, and pazopanib within the World Sarcoma Network. Cancer. (2020) 126:98–104. doi: 10.1002/cncr.32508 31536651 PMC9187112

[19] Fatima J Duncan AA Maleszewski JJ Kalra M Oderich GS Gloviczki P . Primary angiosarcoma of the aorta, great vessels, and the heart. J Vasc Surg. (2013) 57:756–64. doi: 10.1016/j.jvs.2012.09.023 23312835

[20] Poterucha TJ Kochav J O'Connor DS Rosner GF . Cardiac tumors: clinical presentation, diagnosis, and management. Curr Treat Opt Oncol. (2019) 20:66. doi: 10.1007/s11864-019-0662-1 31250250

